# Association between CCC-2 and Structural Language, Pragmatics, Social Cognition, and Executive Functions in Children with Developmental Language Disorder

**DOI:** 10.3390/children8020123

**Published:** 2021-02-09

**Authors:** Clara Andrés-Roqueta, Irene Garcia-Molina, Raquel Flores-Buils

**Affiliations:** Department of Developmental, Educational, Social and Methodological Psychology, Universitat Jaume I de Castelló, 12071 Castelló de la Plana, Spain; imolina@uji.es (I.G.-M.); flores@uji.es (R.F.-B.)

**Keywords:** Children’s Communication Checklist (CCC-2), Developmental Language Disorder (DLD), parents’ reports, formal measures, pragmatics communicative profile, social cognition, executive functions

## Abstract

(1) Background: Developmental Language Disorder (DLD) is diagnosed when the child experiences problems in language with no known underlying biomedical condition and the information required for its correct evaluation must be obtained from different contexts. The Children’s Communication Checklist (CCC-2) covers aspects of a child’s communication related to structural language and pragmatic skills, which are linked to social cognition or executive functions. The aim of this article is to examine parents’ reports using the Spanish version of the CCC-2 questionnaire and its association with different formal assessments related to communication. (2) Methods: 30 children with DLD (3; 10–9 years old) and 39 age-matched (AM) children with typical development were assessed using formal measures of structural language, pragmatics, social cognition, and executive functions. Parents of children with DLD answered the Spanish version of the CCC-2. (3) Results: The performance of children with DLD was lower in all the formal assessments in comparison to AM children. The CCC-2 was significantly correlated with all the direct child assessments, although only formal measures of structural language predicted both the structural language and pragmatics scales of the CCC-2. (4) Conclusions: The CCC-2 answered by parents was consistent with formal assessments in children with DLD, and structural language seemed to be the best predictor of all the subscales.

## 1. Introduction

Developmental Language Disorder (DLD) is diagnosed when the child may be experiencing problems in both the content (semantics) and the form of language (phonology, morphology, and syntax) with no known underlying biomedical condition such as brain injury or autism spectrum disorders, ASD [1]. It is important to highlight that DLD is a heterogeneous condition. Deficits in the acquisition and use of language can be noticed in the reduced vocabulary, limited structure of the sentences, and errors in discourse. The areas commonly affected in DLD (focusing on oral language) are phonology, syntax, word finding and semantics, discourse, verbal learning, and memory, but difficulties also occur in reading and writing. Children with DLD may have problems with language use in social interactions, i.e., pragmatics [2,3]. These problems can have secondary effects in other areas related to social contexts, such as socialization, communication, socioemotional problems, or academic achievement [4,5,6]. According to Bishop and Norbury [7], there is a particular group of children who have fluent, complex, and clearly articulated expressive language, despite showing severe problems in the way the language is used socially. This condition is commonly known as Pragmatic Language Impairment (PLI), or what has more recently been called Social Communication Disorder (SCD; Diagnostic and Statistical Manual of Mental Disorders, DSM-5 [8]; see Davies, Andrés-Roqueta and Norbury [2] for a discussion on whether pragmatic language skills of children with SCD may elucidate sources of pragmatic breakdown in other developmental populations such as autism). With all these difficulties, early diagnosis is crucial because children with DLD have a greater risk of experiencing poor social, emotional, and mental health outcomes [9,10,11,12], which in turn increases the probabilities of them becoming victims of bullying [13].

Information about children’s language and communication development can be obtained in different ways: on the one hand, information about a child’s performance in natural contexts can be provided by parents or teachers and, on the other hand, practitioners also assess children with different formal measures. Standardized tests are important because they are a valid way to find the profile of the individual and any possible deficits. In this sense, both reports and formal assessments are considered an important source of information for establishing a meaningful and contextualized diagnosis [14,15]. Nonetheless, it is worth mentioning that formal assessments require controlled situations, and this therefore represents a contextual limitation for professionals who do not have access to the information about the children’s daily life in more natural contexts, such as at home or school. In these situations, children usually display more natural behaviors, which afford clinical observations and/or assessments [16].

There are different reports to be answered by parents that provide valuable information when children are not yet able to answer for themselves (e.g., Rossetti Infant Toddler Language Scale [17]) or, as in the case of the Social Communication Questionnaire (SCQ, [18]), that are widely available as screening tools; however, the SCQ was designed specifically for detecting risk for ASD, not for DLD. Most language tests or reports assess language structure and content, but are less well suited for evaluating how children use and interpret language or for identifying communication problems, e.g., poor turn-taking or over-literal comprehension [19,20].

In this regard, there is a widely used instrument that employs indirect measures to assess communication behaviors in children: the Children’s Communication Checklist revised (CCC-2). This questionnaire is answered by parents, although it can be completed by any adult who has had regular contact with the child at least 3–4 days per week for at least 3 months, and it provides information about the language and communication profiles in different contexts of interaction (i.e., home or school). Originally, the CCC was designed by Bishop [21] to obtain a quick and easy evaluation measure of children with DLD, predominantly with pragmatic language impairment. Subsequently, the CCC was revised by Bishop in 2003 and standardized with 542 typically developing (TD) children, resulting in the CCC-2. One year later, the CCC-2 was validated with children with diagnoses such as DLD, PLI, and different levels of autism [3].

The CCC–2, with its 70 items spanning 10 subscales, was built to assess different measures of language and communication skills in children from 4 to 16 years of age [22]. Comparisons and results of the CCC-2 have usually been performed based on two overall scores: a general communication composite (GCC), which evaluates children’s communication skills, and a social interaction difference index (SIDI), which specifically assesses children’s pragmatic language. Bishop [22] demonstrated that children with DLD are expected to have difficulty with scales related to structural language, and to do relatively better with scales concerning pragmatics and autistic behavior. Moreover, they would be more likely to obtain a mean GCC below 100. Given the differences in the main composite scores, most studies have employed the CCC-2 as a useful questionnaire that discriminates children with clinical communication problems from typically developing children, as well as differentiating children with DLD from children with pragmatic language impairments. To our knowledge, no research has correlated the subscales with other formal measures or tests.

The CCC-2 was adapted and translated into different languages and went on to become an international tool. The Dutch version of the CCC-2 was used to distinguish the language profiles of children with ASD, DLD, and attention deficit hyperactivity disorder (ADHD) [23]. Moreover, the Dutch version of the CCC-2 added a new pragmatic score called the General Pragmatic Score (GenPragS), which is comparable to the Pragmatic composite score of the original CCC. However, the subscale (D) coherence was added, which was previously classified as a communication skill, but researchers realized that some items referred to pragmatics [20]. The Norwegian version of the CCC-2 successfully differentiated between a group of language-impaired and non-language-impaired children [24]. The Serbian version of the CCC-2 [25] was redefined by Glumbić and Brojčin [26] to obtain three subscales—General Communication Ability, Pragmatics, and Structural Language Aspects—that would distinguish between clinical samples (ASD, ADHD, DLD). The reliability and validity of the CCC-2 was also found in French in Quebec [27], Portuguese [28], and Spanish [29]. Finally, the Spanish version of the CCC-2 was also used to determine whether the CCC-2 was able to identify pragmatic profiles and discriminate between normative and clinical profiles such as significant language difficulties) or Down syndrome [30].

As mentioned above, the CCC-2 has been widely used with the aim of identifying different communication profiles in children with DLD, ADHD, and ASD [21,31,32,33,34,35,36,37,38]. However, the CCC-2 has also been used as a quick screening tool for identifying language disorders in children with sex chromosome trisomies [39], auditory processing disorder [40,41], schizophrenia [42], sleep problems [43], William syndrome [44], and in deaf children with cochlear implants [45]. However, none of these studies have used structural language, pragmatics, social cognition (SC, the ability to attribute mind to others and ourselves) or executive function (EF, a set of cognitive processes that are necessary for the cognitive control of behavior) tests as predictive measures of the CCC-2 scales, although they are considered key predictors of pragmatics [46].

It has been shown that the CCC-2 is a quick and reliable tool that allows professionals to gain a global vision of the problems of language and communication skills that the child may have. In this respect, although they are widely used, formal measures to assess children’s sociocognitive and linguistic profile are usually time-consuming. However, the assessment of pragmatics is difficult and complex [47]. It is a complex area because it is related not only to the use of language in context, but also to SC [46]. Pragmatics and SC are related by definition, given that pragmatic ability underlies the capacity to use and interpret language appropriately in social situations. Studies using screening instruments and conversational analysis have reported pragmatic deficits among children with DLD [3]. Similarly to ASD (but with fewer empirical studies conducted in the area), research based on experimental tasks has described problems in specific areas of pragmatics such as understanding figurative language [48,49,50], sensibility to conversational maxims like “quantity” [51,52] and other Gricean maxims [53], using the preceding context to resolve ambiguous utterances and in narrative production [54,55], or understanding graphic humor [56]. Their pragmatic skills are usually in keeping with the levels of their structural language, as they perform as well as younger TD children matched for language level in experimental tasks [51]. Moreover, their level of SC also matters for these pragmatic problems when the tasks are socially oriented [57].

One of the most widely used SC measures is the Strange Stories task [58]. This task allows pragmatic impairment to be detected in children with DLD and ASD, because children must grasp the speaker’s real intention by understanding his/her utterance (sometimes non-literal) and contextual aspects concerning the communicative situation [46]. The Strange Stories task involves stories about sarcasm, irony, and white lies, among other things. For example, in the ironic story, a character uses an utterance (“Well, that’s very nice, isn’t it!”) to remark that a person is being rude. Thus, the participant must use his or her structural language competence to understand the literal meaning, and may understand the context and pragmatic maxims for the “hidden” intended meaning [51,57,59]. Interestingly, there is no empirical evidence from studies using the CCC-2 pragmatic subscales and establishing relationships with SC tasks.

Likewise, very little work has explored the relationships between pragmatics and EF in children with DLD. The rules of conversation change depending on the context in which they occur, and therefore they allow us to adapt our speech flexibly to the dynamic demands of the context by being flexible, tight, and effective [60]. Furthermore, maintaining a coherent reciprocal conversation requires paying attention and remembering what our speaker is saying (therefore using our attention and working memory skills). At the same time, we also need to inhibit excessive talking and ensure that our contribution is relevant (thus using our inhibition, organizing, monitoring, and planning skills) (see Green et al. [61] for a review).

The novelty of the present study is the fact that it has broken the CCC-2 down into simple scales (structural language, pragmatics, and autistic subscores) for associations and predictions with formal measures of structural language, pragmatics, SC, and EF. The present paper thus aims to examine whether parental information provided by the CCC-2 questionnaire is significantly associated with formal measures of structural language, pragmatics, EF, and SC in children with DLD. In this regard, the following hypotheses are stated:

**Hypotheses** **1** **(H1).** *First, the responses given by the parents are expected to show the language and communication problems of their children according to their age.*

**Hypotheses** **2** **(H2).** *Second, the CCC-2 scales (structural language, pragmatics, and autistic subscores) are expected to be associated with clinical-related tests (i.e., the scales of structural language will correlate to phonetics, syntax, and vocabulary tests; the pragmatic and autistic scales will correlate with SC, EF, and pragmatics) [51].*

**Hypotheses** **3** **(H3).** *Finally, formal measures are also expected to predict the CCC-2-related scales (i.e., the scales of structural language will correlate with the phonetics, syntax, and vocabulary tests; the pragmatic and autistic scales will correlate with SC, EF, and pragmatics).*

## 2. Materials and Methods

### 2.1. Participants

#### 2.1.1. DLD Group

The DLD sample comprised 30 Spanish-speaking children (8 girls and 22 boys) diagnosed with DLD with ages between 3;10 and 9;0 years (mean age 70.50 months, range = 46–108). Children came from different ordinary schools in Spain.

- Diagnosis of DLD: Children with DLD had an updated diagnosis by a qualified educational psychologist from the ordinary schools where the sample was recruited. All these psychologists from the different schools belonged to the same local health services, and so they followed the same diagnostic protocols, thereby ensuring that the criteria used for all these diagnoses were homogeneous. In this regard, their records confirmed that the children had substantial language disability as the main cause for receiving speech and language therapy, while presenting a typical nonverbal intelligence based on standardized language and cognitive tests. Moreover, participants were recruited for the DLD group if they were native speakers of Spanish receiving speech and language therapy in the school at the time of the study; had language difficulties (discarding possible auditory disorders, neuro-sensory and intellectual disability); and finally, their clinical record had to be free of any medical condition that was likely to affect language, such as a diagnosis of ASD. Additionally, DLD condition was confirmed by the research group. The research team assessed each selected participant using two standardized grammar measures: on the one hand, Comprensión de Estructuras Gramaticales [62], and on the other hand, a Memory subtest on the Evaluación del Lenguaje Infantil, which is a sentence repetition task that measures expressive language ability and short-term auditory memory (ELI; [63]), both of which have been reported as a valid formal measure in the diagnosis of DLD (e.g., [64]). Participants were recruited only if they scored one standard deviation below the mean in at least one of these two tests. This threshold has been used in similar papers to conduct in-depth studies in children with DLD [52]. Moreover, Raven’s Progressive Matrices revised version [65] was used to ensure that the children’s IQ was within the normal range (above the 15th percentile). In this sense, the DLD group obtained a mean of 28.57 (SD = 72.04, range = 25–99).

#### 2.1.2. Chronological Age-Matched (AM) Group

The AM sample comprised 39 Spanish-speaking children (11 girls and 28 boys) aged between 3;7 and 9;00 years old (mean age 70.50 months, range = 43–109). The children came from the same ordinary schools as those in the DLD group. They were age- and gender-matched to children with DLD within ± 3 months of age. Furthermore, the educational psychologists were consulted to ensure that the children were not receiving speech and language therapy at the school when the study was being conducted or prior to it.

The AM group was introduced as a control to be compared with the DLD group on formal measures of structural language, pragmatics, SC, and EF. However, it was not used to compare the CCC-2 scores because: (1) This was not one of the main aims of the present study; and (2) The CCC-2 provides centile scores to compare a child with a normative mean, but the other measures (language, SC, and EF) are used with raw scores to be introduced in the correlational and predictive analyses. Therefore, the AM group was used to show whether the DLD group also had age-impaired performance on those measures.

### 2.2. Measures

#### 2.2.1. Parent Reports

##### Children’s Communication Checklist (CCC-2)

Parents of the participants with DLD answered the Spanish version of the CCC-2 (CCC-2—Spain/Spanish—Version 2 of 22 Jun 2012—MAPI Institute, provided by Pearson to the research group). The CCC–2 is used for children between the ages of 4;0 and 16;11 [19]. It is a 70-item questionnaire that caregivers rate using a 4-point numeric frequency scale ranging from 0 to 3, where 0 = less than once a week (or never); 1 = at least once a week, but not every day (or occasionally); 2 = once or twice a day (or frequently); 3 = several times (more than twice) a day (or always). It measures ten scales of communication, each with seven items: A. Speech, B. Syntax, C. Semantics, D. Coherence, E. Inadequate Initiation, F. Stereotyped Language, G. Context, H. Non-verbal Communication, I. Social Relations, and, J. Interests. The first four scales (A–D) measure structural language aspects; the four intermediate scales (E–H) measure pragmatic aspects, and the last two areas (I–J) measure autistic behavior. Internal consistency values between 0.66 and 0.80 and inter-rater reliability between parents and teachers ranged from 0.16 to 0.79 for the CCC-2.

Table 1 shows, by way of example, one item from each of the communication aspects measured in the CCC-2.

The CCC–2 produces the General Communication Composite (GCC) and the Social Interaction Difference Index (SIDI).

The GCC is a norm-referenced standard composite score that reflects overall communication skills (M = 100, SD = 15), and it is used to identify clinically significant communication problems. It is computed by adding scaled scores from Scales A through H.

The SIDI is an index score that reflects the difference between the structural language scales (A, B, C, and D) and the pragmatic language scales (E, H, I, and J), so it provides an index of mismatch between structural language skills and pragmatic and social skills [50]. SIDI scores ranging from −10 to 10 are considered typical, and scores within this range were obtained by 90% of the CCC–2 normative sample. Scores ≥ 11 suggest that speech/syntactic/semantic skills are deficient and relatively poorer than pragmatic skills, whereas scores ≤ −11 suggest that pragmatic language skills are deficient and relatively poorer than syntactic/semantic skills; this profile is associated with ASD [22].

However, in the present paper, the SIDI score was not used for correlations and the regression analysis, because it is used to ascertain the nature of an identified communication impairment and should, therefore, usually only be taken into account when the GCC is less than 55 (which ranged from 30 to 100 in the DLD in the present study, see Section 3) [19,66]. Moreover, other scores can be formed for more information by adding scales, such as structural language skills (A + B + C + D) [33,67,68]. Geurts [23] also proposed a second composite score that can be calculated as the sum of the scales D–H (GenPragS). This index was also used in the present study due to the fact that item (D) Coherence mixed aspects of structural language and also pragmatic skills.

In this regard, the three main subscales *Structural language* (A + B + C + D, scores ranging from 0 to 28), *Pragmatic* (D + E + F + G + H, scores ranging from 0 to 35), and *Autistic* (I + J, scores ranging from 0 to 14) composite scores were created for the correlational and predictive analyses by adding up the raw scores. It must be highlighted that the raw scores on the CCC-2 indicate the amount of difficulty on the different scales (in contrast to the scaled scores).

#### 2.2.2. Formal measures

- Non-verbal reasoning

Raven’s Colored Progressive Matrices scale was administered in order to have a non-verbal reasoning score for each DLD and AM participant [65]. Raw scores in this test range from 0 to 36. The standardization study yielded a value of 0.80 in test-re-test reliability [69].

- Structural language

*Phonetics: Phonetics subtest* from Evaluación de Lenguaje Infantil, ELI [63]. The phonetic subtest measures the level of articulation, the ability to imitate phonemes, and the difficulties to pronounce words containing “he studied” phonemes in initial, middle or final position. Children pronounced these words that could be represented as images or also by imitating adults’ pronunciation. As the phonetics subtest is qualitative, the scoring has been processed emphasizing the type of errors that the child exhibited in the following way: No problems = 0; With substitutions in simple structures of (Consonant–Vowel) = 1; With mistakes in complex structures (Consonant–Consonant and Vowel–Vowel) = 2; With all the types of mistakes = 3. Scores were based on how complex the phonetic problems were: the more complex the problems were, the higher the score was. The maximum score was 3.

*Receptive grammar:* Comprensión de Estructuras Gramaticales, CEG [62]. This is an instrument designed to evaluate the participant’s capacity to understand different types of grammatical constructions (grammatical comprehension) through drawings and sentences, with sentences of varying lengths and degrees of complexity. The CEG is a Spanish adaptation of the Test for Reception of Grammar, TROG [70], which assesses English grammar comprehension in children. Raw scores in this test range from 0 to 80.

*Expressive grammar: Sentence recall subtest* from Evaluación de Lenguaje Infantil, ELI [63]. This subscale was used to evaluate grammatical expressive skills. It measures the average sentence length and short-term auditory memory. Raw scores in this subtest range from 0 to 10.

*Receptive vocabulary: Receptive Vocabulary subtest* from Evaluación de Lenguaje Infantil, ELI [63]. This subscale measures the level of knowledge of vocabulary and receptive vocabulary. There are 30 sheets of increasing difficulty (from more concrete to more abstract semantic fields), and the child should point to the drawing that was asked for. Raw scores in this subtest range from 0 to 30.

*Expressive vocabulary: Expressive Vocabulary subtest* from Evaluación de Lenguaje Infantil, ELI [63]. This subscale measures access to vocabulary, expressive vocabulary, semantic knowledge, and information. It consists of 30 drawings, which the child must name (words from the close context in growing difficulty). Raw scores in this subtest range from 0 to 30.

*Structural language composite score:* These last five language measures were used to examine structural language as a complex construct. However, as these measures use different scoring ranges, a composite score was created. Raw scores, rather than standardized scores, were used because they are a direct indicator of how many correct responses each child achieved in each test. For phonetics, we created an inverse variable. The measures were weighted equally and combined to form the language composite score. This approach was taken to address issues arising from different scaling within language measures and between the other non-standardized measures used in the study. Specifically, the language composite variable was obtained by adding together all five linguistic raw scores. The final score ranged from 0 to 100, with each language measure representing 1/3 of the new composite score (1/3 phonetics, 1/3 grammar and 1/3 vocabulary).

- Pragmatics

The pragmatics subscale from the ELI battery [63] was used to obtain a formal assessment of functional communication. This subscale has both *receptive* items related to gesture–speech integration (e.g., the examiner tells the child a sentence and the child must decide if there is a discrepancy between statements and gestures), and *expressive* items related to figurative language understanding and politeness (e.g., the examiner asks questions about politeness, like “What do you say when somebody gives you a present?” or idiom questions like “What does ‘you’re a pig’ mean?’).

*Pragmatic composite score:* The two pragmatic measures (expressive and receptive) were used to examine pragmatics by adding the two scores (as stated in the ELI test). Raw scores in this subtest range from 0 to 14.

*A note about the use of ELI: This test has two versions (Catalan and Spanish) and it can be administered in both languages. Nevertheless, the participants of the present study were assessed with the Spanish version since their mother tongue was Spanish. Furthermore, it must be noted that the ELI test is usually used for children from 3 to 6 years old. However, practitioners also use it for older children with DLD. In this sense, the research group selected ELI because the DLD sample included children from 3;10 to 9;00 years old, and so it allowed us to test smaller children (e.g., the Batería de Lenguaje Objetiva y Criterial - BLOC - test starts at 5 years old, and the Prueba de Lenguaje Oral Navarra - PLON - covers similar age ranges as ELI). Moreover, regarding the aims of the study, we were interested in raw scores to carry out the correlational and predictive studies.

*A note on the reliability of ELI and CEG

The ELI test is widely used to assess children’s language. ELI has adequate Psychometric properties: Validity, correlation values: ELI Comprehensive Vocabulary–Peabody (*r* = 0.75); ELI Expressive Vocabulary–Kaufman Brief Intelligence Test (K-BIT) (*r* = 0.85); ELI Sentences– Wechsler Preschool and Primary Scale of Intelligence (WPPSI) Sentences (*r* = 0.51) (K-BIT [71]; WPPSI [72]). Reliability, split-half reliability and Test-Retest were used: Expressive-Vocabulary (0.83), Sentences (0.71), and Comprehensive–Vocabulary (0.70). 

CEG has adequate Psychometric properties: Reliability: 0.91; Validity, correlations values: CEG-Peabody (*r* = 0.809, *p* = 0.00); and CEG– Illinois Test of Psycholinguistic Abilities (ITPA) (*r* = 0.644, *p* = 0.00) (Peabody, [73]; ITPA, [74]). Discrimination: more than half of the elements provide a discrimination index greater than 0.3 among subjects with higher scores and lower scores in the test [75].

- Social cognition

*Strange Stories* [58]. The aim of this task is to assess the understanding of other people’s communicative intentions when non-literal language is used. Six of the original stories were used: pretense, lie, white lie, irony, joke, and idiom. The participants saw and listened to the Strange Story, and then, based on the reply, the scores were calculated as follows: 0 = inappropriate without mentalist aspects; 1 = inappropriate with mentalist aspects—distinct intention; 2 = correct with explicit aspects; and 3 = mentalist intention correct with expected intention [51]. Raw scores in this test range from 0 to 18. Individual mental state stories exhibited moderate to strong inter-rater reliability (M κ = 0.82, range = 0.79 ≤ κ ≤ 0.85, all *p* < 0.01), as did individual control stories (M κ = 0.84, range = 0.74 ≤ κ ≤ 0.95, all *p* < 0.01) [76].

- Executive Functions

The *Matching Familiar Figures Test* (MFFT; [77]) was administered to measure sustained attention and inhibitory skills. The MFFT consists of 12 items where the children were shown a picture (person or object) and six similar stimuli and the children were required to pick the picture that was identical to (e.g., that matched) the person/object given. Two variables were provided: the total number of errors committed until the correct one was found (*sustained attention*), and the mean latency prior to the first response (*response latency*). Correlations were 0.91 for latency and 0.89 for errors [77].

*EF composite score*: The two measures (sustained attention and response latency) were used to examine executive functions. Nevertheless, as these measures have different scoring ranges, a composite score was created. For sustained attention, we created an inverse variable because it indicates the number of errors committed (and not capacity), whereas response latency indicates the number of seconds before the first response (capacity to inhibit first response). The measures were weighted equally and combined to form the EF score. The final score ranged from 0 to 100 with each EF measure representing 1/2 of the new composite score 1/2 sustained attention, 1/2 response latency).

### 2.3. Procedure

Permissions were requested from the Regional government and school authorities to select the ordinary schools that children with DLD attended. Four schools agreed to take part in this study. Parents were then informed about the aims of the study, and written consent to participate was obtained.

Each child was assessed with the study instruments by the research group during two 40 min sessions (approx.) in a quiet room provided by the school. Tasks were administered individually, in random order. In parallel, parents were interviewed individually by the research group, and they completed the CCC-2 in the same schools, one hour before they picked their children up from school.

The original sample included in this study included 35 parents. One was lost because those parents failed to answer the questionnaire and four of them did not pass the Consistency check, which shows that the respondent has understood how to complete the CCC-2 with regard to positively and negatively formulated items [19].

### 2.4. Data Analysis

Data analysis was conducted using the statistical package SPSS (version 27). When the sample was subdivided into two groups (DLD and AM), the data failed the Shapiro–Wilk test of normality, showing several unequal variances across groups for the different scores. Moreover, there was a different number of participants in each group. Therefore, Mann–Whitney U (two-tailed, significance threshold of 0.05) was used to examine differences between groups on key measures. Effect sizes of group comparisons were calculated using *r* with the formula: *r* = (*z*)/(√N), because according to Fritz, Morris, and Richler [78], when between-group comparisons are performed with Mann–Whitney U tests, size effects must be calculated using “*r*” and not “d”, where a value of 0–0.1 is considered a small effect; 0.2–0.4 is considered a medium effect; and 0.5–1 is considered a large effect.

Moreover, zero-order nonparametric correlations (Spearman) between key measures were conducted within the DLD groups. Finally, in order to further investigate the contribution of age, structural language skills, social pragmatics, linguistic pragmatics, and executive functions on the CCC-2 scores, a hierarchical linear regression analysis was conducted for the whole clinical sample (*n* = 30), and a bootstrapping method was implemented using 2000 bootstrap samples to derive robust estimates of standard errors, confidence intervals, and *p* values of the regression model.

It must be stated that the AM group was only used to compare the DLD group in the scores from the formal measures, but not in the CCC-2. This was because, as explained in the Participants section, the main aim of the present study was not to see the association of the CCC-2 with the related variables in typically developing children. In this sense, correlations and regressions are only carried out within the group of children with DLD and not among the AM children.

## 3. Results

### 3.1. Descriptive Statistics and between-Group Comparisons on Formal Measures

Table 2 reports the descriptive statistics of the four groups for grammar, age, structural language, pragmatics, SC, and EF formal measures.

The Mann–Whitney U-test showed that the groups did not differ in age (*U* = 560.500, *p* < 0.001, *r* = 0.036) and small effect sizes were observed. Moreover, a Chi-squared test showed that groups did not differ according to gender (χ^2^ = 0.20, *p* = 0.887).

As regards the formal measures (raw scores) of structural language, pairwise comparisons revealed that the DLD and AM groups differed in all the measures: phonetics, expressive grammar, receptive grammar, receptive vocabulary, expressive vocabulary, and also in the structural language composite score. Large-size effects were observed for grammar and phonetics measures, as well as for the composite score, whereas medium-size effects were observed for vocabulary measures.

Regarding pragmatic scores, between-group comparisons revealed differences between the DLD and the AM groups: receptive pragmatics, expressive pragmatics, and pragmatics composite score. Large-size effects were observed for all cases.

Regarding the social cognition measure, between-group comparisons revealed differences between the DLD and the AM groups, and large-size effects were observed.

Finally, turning to the executive function measures, again, between-group comparisons showed that the AM group achieved a significantly lower performance than the DLD group: sustained attention, latency of response, and EF composite variable, and large- and medium-size effects were observed.

### 3.2. Descriptive Statistics on CCC-2

Table 3 reports the descriptive statistics on the CCC-2 for the DLD group.

As regards the GCC score (scaled scores), the DLD group obtained a mean of 67.53, which is almost the cutoff range of language impairments.

As regards the SIDI index (scaled scores), the DLD group obtained a mean of 10.07, which is a value that is included within normal limits (the normal range of SIDI scores is from −10 to 10).

As regards Structural language (scaled scores), the DLD group obtained a mean of 28.57; as regards Pragmatics (scaled scores), the DLD group obtained a mean of 28.57; and as regards the Autistic index (scaled scores), the DLD group obtained a mean of 18.53.

Individual scores demonstrate that the DLD group showed better performance on scales E, F; then on D, G, H; then I and C; and the lowest means were obtained on A and B.

### 3.3. Correlations between CCC-2 Scores and Age, Structural Language, Linguistic Pragmatics, Social Pragmatics and Executive Functions

Zero-order nonparametric correlations (Spearman) between key measures are presented in Table 4.

First, the three scores derived from the CCC-2 proved to be positively and strongly correlated: Structural Language–Pragmatics (*p* < 0.001); Structural Language–Autistic index (*p* = 0.001); Pragmatics–Autistic index (*p* = 0.003).

Strong and negative correlations were observed between Structural Language measured with the CCC-2 and age (*p* < 0.001), the structural language composite score based on formal measures (*p* < 0.001), and the executive functions composite score (*p* < 0.001). Moreover, a medium and negative correlation was observed with pragmatics (*p* = 0.014). However, it was not correlated with social cognition (*p* = 0.055). The correlations are negative because raw scores on the CCC-2 indicate the degree of difficulty on the different scales (in contrast to scaled scores).

For the Pragmatic measure of the CCC-2, a negative and non-significant correlation was observed with age (*p* = 0.219). Medium and negative associations were observed with formal measures of structural language (*p* = 0.014), pragmatics (*p* = 0.028), social cognition (*p* = 0.011), and executive functions (*p* = 0.005). Again, the correlations are negative because raw scores on the CCC-2 indicate the degree of difficulty on the different scales.

Finally, for the Autistic measure of the CCC-2, a negative and non-significant correlation was observed with formal measures of age (*p* = 0.139), pragmatics (*p* = 0.246), and social cognition (*p* = 0.134). Medium and negative associations were observed with formal measures of structural language (*p* = 0.014) and executive functions (*p* = 0.010). Again, the correlations are negative because raw scores on the CCC-2 indicate the degree of difficulty on the different scales.

### 3.4. Predictive Analysis of the CCC-2 Scores of Structural Language, Pragmatics and Autistic Indexes

As shown in the correlation analyses, several variables were correlated in the DLD group, making it difficult to identify the independent contribution each of them makes to the CCC-2 measures. To further investigate the contribution of these variables, three hierarchical linear regression analyses were conducted for the DLD group *(n* = 30).

Structural Language (CCC-2: A + B + C + D), Pragmatics (CCC-2: D + E + F + G + H), and Autistic score (CCC-2: I + J) were the outcome variables in the regression, and five predictor variables were entered in the following order (Table 5 and Table 6): age was entered first, as raw scores had been used, and also because there was an important age difference between some participants in the sample (ranging from 3 to 9 years old). The Structural Language composite score (formal measure) was entered next, because structural language deficits are fundamental factors for the pragmatic deficits observed in children with DLD (e.g., [51]). Pragmatic and social cognition scores were entered after structural language to investigate whether they make a specific contribution to each CCC-2 score when structural language skills have been taken into account. Finally, the executive function composited variable was introduced in the final stage, to check whether other features related to sustained attention and latency of response are relevant.

For Structural Language (CCC-2), the general model was significant and accounted for 64% of the variance, F (5, 23) = 8.225, R^2^ = 0.641, *p* < 0.001 (see Table 5). Higher Structural Language scores on the CCC-2 were negatively and significantly associated with Age, which explained 37% of the variance, and higher structural language scores of formal measures, which explained 23% of the variance. No single association was found with the formal assessments on pragmatics, social cognition, or executive functions.

For Pragmatics (CCC-2), the general model was significant and accounted for 37% of the variance, F (5, 23) = 2.692, R^2^ = 0.369, *p* = 0.047 (see Table 6). Higher Pragmatic scores on the CCC-2 were negatively and significantly associated with structural language formal measures, which explained 16% of the variance. No single association was found with the formal assessments on age, pragmatics, social cognition or executive functions.

Finally, for the Autistic index (CCC-2), the general model was not significant: F (5, 23) = 1.296, R^2^ = 0.220, *p* = 0.300. In this sense, no single association was found with formal measures of age, structural language composited score, pragmatics, social cognition or executive functions.

## 4. Discussion

The present study attempted to determine whether the Spanish version of the CCC-2, applied to parents of children with DLD, agrees with clinical information when linguistic, pragmatic, EF, and SC areas are assessed through direct measures. Furthermore, this information from parents allows us to better understand the children’s problems, in a more detailed and contextualized way than with only the results of direct measures.

Regarding the first hypothesis, the responses given by parents were expected to allow us to better understand the problems of language and communication that children with DLD have. First, all the formal measures discriminated between the two groups (DLD–AM), demonstrating that the performance of those with DLD was lower than that of their AM peers in structural language, pragmatics, SC, and EF measures, in line with previous studies (for structural language and pragmatics: [46,49,50,51,52,53,54]; for SC [46,51,57,59]; and for EF [60,61]). Also, regarding previous findings using other versions of the CCC-2 (e.g., Norwegian sample), a cutoff at or below a scaled score of 64 on the GCC was selected to identify children with language impairments [24]. Our sample was closer to the cutoff, which would corroborate their problems in structured language. Moreover, taking a closer look at the scales, their lower means were found in (A) Speech and (B) Syntaxis. However, the children with DLD obtained a positive score (within normal limits) in the SIDI, indicating no disproportionate pragmatic impairments according to their structural language abilities. Again, a more detailed observation shows that the best scores of the children with DLD were found on the pragmatic scales (E > F > D > G > H).

With regard to Hypothesis 2, the CCC-2 scales were associated with the different clinical-related tests (formal measures). In this sense, Structural Language (CCC-2) was correlated with age, structural language, pragmatics, and EF (but not with SC); Pragmatics (CCC-2) was correlated with structural language, pragmatics, SC, and EF (but not with age); and the Autistic index (CCC-2) was correlated with structural language and EF (but not with age, pragmatics, or SC). All the CCC-2 correlations were in line with what was expected for Structural Language and Pragmatic scores, but not for the Autistic index, which was expected to also correlate with SC. SC and Pragmatics would go hand in hand for the complete understanding of the contextual aspects around the communicative situation [46]. In the same sense, EF would seem to be crucial to remember the interlocutor’s information, monitoring, and planning the speech or to know when it would be better to initiate a conversation [60,61].

It is important to note the significant correlations found on comparing parents’ opinions and the clinical measures. In this regard, it was found that parents’ and professionals’ criteria ran in the same direction, which is useful for detecting and recognizing their children’s language and communication handicaps. It should be highlighted that parents’ information would be useful not only in the aspects referring to the structural language (phonology, morphosyntax, and semantics), but also in the pragmatics areas, which are less easily measurable in the clinical setting (inadequate initiation, context, non-verbal communication). Thus, it is concluded that the parents’ opinions agree with the clinical assessments.

Together, these findings identify structural language, pragmatics, SC, and EF as key skills for communication in the DLD group.

Finally, in Hypothesis 3, results corroborated the idea that formal measures predicted the CCC-2 scales related to structural language and pragmatics. On the one hand, age and structural language predicted the structural language index (CCC-2), which makes sense as the errors in phonetics, syntax and vocabulary affect the structural language index (CCC-2), and, in the same line, the formal measures of structural language. On the other hand, structural language predicted the Pragmatic Index (CCC-2), but not age or formal measures of pragmatics, EF, and SC. Again, this could be explained by the need to use the structural language competence to understand the literal meaning at first to fully comprehend the hidden intention or the context where the utterance is made [51,57,59]. Finally, none of the formal measures predicted the Autistic Index, which could be explained by the fact that the sample consisted only of children with DLD (non-autistic children) with specific difficulties in structural language and pragmatics, rather than SC and EF.

Specifically regarding pragmatic competence, between-group comparisons confirmed that children with DLD faced significant difficulties on the formal measures (including structural language, pragmatics, SC, and EF tasks) compared with their AM peers. However, difficulties with pragmatics were in keeping with the participants’ structural language (but not SC or EF). This issue emphasizes the role of structural language in pragmatic development for children with DLD, obtaining similar findings to those of previous research (e.g., [2,49,50]; for a complete review, see [46].

As a limitation, we must be cautious with our results. First, a larger sample size would be needed in future studies to be able to conduct a meticulous study of the overlap between structural language, SC, EF, and pragmatics. Moreover, most of the participants in the present study are still developing some of the skills measured, so the results of the present study are applicable to children with DLD from 4 to 9 years old, but not for older ones. In this sense, most of the studies cited have assessed samples of older children with DLD, and pragmatic problems are perhaps more salient and best predicted by SC or EF, instead of only structural language. Furthermore, the Spanish language entails greater difficulties in morphological inflections compared to English (gender and number concordance, or verb conjugations). Other cultural influences that are more related to pragmatic aspects could exert an influence (e.g., accepted distance between interlocutors, improper initiation, or turn-taking behavior accepting more interruptions). Finally, there is a need for further research on the exact characterization of the different pragmatic skills and whether they depend on the phenomenon under study (e.g., figurative understanding, gesture–speech integration or understanding irony), on the structural aspects linked to understanding the linguistic context, on the SC strategies of the listener, or on the EF skills to maintain and initiate communication with other people [46,51]. In this respect, after examining the results, training of structural language skills seems essential for the development of pragmatic skills in children with DLD between 4 and 9 years of age. Furthermore, it is important to remember that the pragmatics subtest of ELI may not be enough to measure the child’s pragmatic competences in an exact and complete way. In fact, it is a quick measure that can give us an initial idea. However, this subtest cannot be compared with pragmatic tests in which the evaluation is based on natural observation, through the behavior of the participants in a specific context. In Spanish, professionals and practitioners need more complex formal batteries to assess pragmatic competences in a comprehensive way.

## 5. Conclusions

In sum, the results of the present study responded to our main goal. The parents’ reports on the CCC-2 were consistent with the professionals’ formal evaluations. Another conclusion that could be drawn is that the extent and underlying causes of general communication difficulties of children with DLD correlate not only on the children’s competence with structural language and pragmatics, but also on SC and EF. Nevertheless, structural language seems to be the best predictor of all the subscales measured with the CCC-2.

It is important to highlight that, on the one hand, parents’ responses have been seen as important and complementary cues to complete the important information about children’s communicative skills in different contexts. Furthermore, it should be highlighted that the CCC-2 is an informative, fast, and cost-effective tool to measure and anticipate language impairments in preschool and school-age children. Since parents can participate, clinicians can have access to the children’s daily life in a more natural context than in a clinical setting. It should be added that, according to our data, the information provided by the parents seems to be precise in structural language aspects (the most visible in communication), but they do not seem to be aware of the actual pragmatic implications/difficulties, as some pragmatic skills develop during later childhood. In this regard, clinicians should make parents aware of these difficulties and help them with guidelines for intervention in pragmatic aspects. Future research should investigate the use of these kinds of tools with larger samples, while also adding more sophisticated items to address pragmatic components, given that they are more difficult to evaluate in designs involving children.

Moreover, as demonstrated in the present study, structural language skills affect the general communication of a child with DLD. Therefore, focusing only on pragmatics without taking into account other structural language will not reveal the actual communication needs of a child with DLD and might result in treatment goals that are too tightly defined [34].

Consequently, multi-disciplinary assessments of the communication profile of a child with DLD are necessary to design an adapted and individualized intervention (e.g., include formal assessments together with parents’ reports on aspects of communication). Moreover, it seems very important to include structural language contents in interventions that aim to improve pragmatic competence [51]. However, it would also be important to include aspects of SC (e.g., regarding the understanding of a speaker’s intention) and EF (e.g., regarding attention to cues from context, or impulsive or quick responses to the speaker) to improve other social communication skills that have not been addressed with the CCC-2 (e.g., understanding irony). Early diagnosis of pragmatic difficulties is crucial due to the fact that children with DLD are at greater risk of experiencing poor social, emotional, and mental health outcomes [9,10,11,12], which increases the probabilities of them being the victim of bullying [13].

## Figures and Tables

**Table 1:** Examples of items from the communication aspects measured in the Children’s Communication Checklist-2 (CCC-2).

Aspects of Communication	Sample Items
A. Speech	He/she speaks fluently and clearly, producing all speech sounds accurately and without hesitating.
B. Syntax	He/she produces long and complicated phrases like: “When we were in the park I went to see the ducks” or “I saw that man standing on the corner”.
C. Semantics	He/she uses words that refer to classes of objects, rather than specific things; e.g., talks about the table, chair and drawers as “furniture”, or calls bananas, apples and pears “fruit”.
D. Coherence	It is difficult to know whether he/she is talking about something real or something invented.
E. Inappropriate initiation	He/she talks repeatedly about things that no-one is interested in.
F. Stereotyped language	When he/she answers a question, he/she provides sufficient and relevant information, without being overly precise if it is not necessary.
G. Context	His/her ability to communicate is different according to the situation. He/she may not have any trouble talking one-on-one with a familiar adult, but may find it difficult to express him/herself with a group of children of his/her own age.
H. Non-verbal communication	He/she doesn’t look at people when he/she talks to them.
I. Social Relations	He/she hurts or disturbs other children without realizing it, unintentionally.
J. Interests	He/she leads the conversation towards his/her favorite topics, even when others are not interested.

**Table 2:** Descriptive statistics of Developmental Language Disorder (DLD) and chronological Age-Matched (AM) groups and between-group comparisons on formal measures.

	DLD (*n* = 30)		AM (*n* = 39)		*U*	*p*	*r*
	M (SD)	Range	M (SD)	Range			
Age and Gender							
Months	70.50 (17.86)	46–108	68.62 (14.26)	43–109	560.5	0.767	
Gender (M:F)	22:08		28:11:00		-	-	
Structural language measures							
Phonetics (range: 0–3)	1.07 (0.91)	0–3	0 (0)	0–0	156	0	0.761
Receptive grammar (range: 0–80)	50.43 (10.62)	17–68	64.69 (7.56)	48–77	146	0	0.682
Expressive grammar (range: 0–10)	5.13 (1.83)	2–8	7.97 (1.31)	4–9	126	0	0.64
Receptive vocabulary (range: 0–30)	21.03 (6.22)	5–30	23.92 (4.91)	10–29	390	0.018	0.285
Expressive vocabulary (range: 0–30)	18.47 (6.31)	7–27	22.64 (6.20)	10–30	351	0.005	0.341
Structural language	53.71 (16.62)	14.54–75.53	75.62 (6.24)	60.62–83.7	89	0	0.722
Pragmatics							
Receptive pragmatics (range: 0–6)	4.8 (1.32)	1–6	5.59 (0.91)	2–6	332.5	0.001	0.414
Expressive Pragmatics (range: 0–8)	2.8 (1.91)	0–8	5.10 (1.77)	1–8	213.5	0	0.547
Pragmatics (range: 0–14)	7.6 (2.66)	3–14	10.69 (2.33)	5–14	221.5	0	0.533
Social cognition measure							
Speaker’s intention (range: 0–18)	4.8 (3.68)	0–12	10.87 (3.87)	1–16	152.5	0	0.632
Executive function measures							
Sustained attention	22.14 (12.58)	7–53	15.74 (7.14)	4–32	405	0.046	0.24
Response latency	7.3 (5.71)	1.71–20.30	15.40 (12.57)	2.82–75.15	260	0	0.456
Executive function	33.53 (14.64)	2.28–56.75	44.67 (12.56)	20.3–82.46	323	0.003	0.362

Note: *Raw scores* on all measures; *Age* = chronological age (months); *Structural Language* = Language composite score; *Pragmatics* = Pragmatic composite score; *Executive function* = Executive function composite score.

**Table 3:** Descriptive statistics of Developmental Language Disorder (DLD) participants on Children’s Communication Checklist-2 (CCC-2) scores.

	DLD (*n* = 30)	
	M (SD)	Range
GCC score	67.53 (16.71)	30–100
SIDI index	10.07 (9.28)	−7–39
Structural Language (A + B + C + D) scaled score	28.57 (8.07)	14–49
Pragmatics (D + E + F + G + H) scaled score	47.93 (12.45)	28–73
Autistic index (I + J) scaled score	18.53–5.91	11–35
A. Speech	6.47 (3.39)	0–13
B. Syntax	5.6 (3.05)	0–12
C. Semantics	7.53(2.36)	4–15
D. Coherence	8.97 (3.2)	5–16
E. Inappropriate initiation	11.37 (4.06)	4–21
F. Stereotyped language	10.37 (3.38)	5–16
G. Context	8.50 (3.19)	4–20
H. Non-verbal communication	8.73 (3.99)	3–21
I. Social relations	7.37 (4.09)	2–21
J. Interests	11.17 (4.55)	1–22
Structural Language (A + B + C + D) raw score (range: 0–28)	21.37 (12.54)	2–52
Pragmatics (D + E + F + G + H) raw score (range: 0–35)	21 (12.14)	5–45
Autistic index (I + J) raw score (range: 0–14)	8.20 (3.75)	3–18

Note: scaled scores on all measures except for the last three measures.

**Table 4:** Zero-order nonparametric Spearman correlations between formal measures and Children’s Communication Checklist-2 (CCC-2) scores within the Developmental Language Disorder (DLD) group (*n* = 30).

	Age	St. Lang	Prag	SC	EF	St. Lang CCC-2	Prag CCC-2
Age							
St. lang	0.624 **						
Prag	0.382 *	0.557 **					
SC	0.292	0.434 *	0.384 *				
EF	0.563 **	0.593 **	0.552 **	0.250			
St. lang CCC-2	−0.705 **	−0.703 **	−0.445 *	−0.354	−0.685 **		
Prag CCC-2	−0.231	−0.442 *	−0.401 *	−0.457 *	−0.510 **	0.663 **	
Aut CCC-2	−0.276	−0.444 *	−0.219	−0.280	−0.472 **	0.568 **	0.527 **

Note 1: * *p* < 0.05; ** *p* < 0.01; Note 2: all scores are raw scores; Note 3: Age = chronological age (months); St. lang = Language composite score (formal measures); Prag = Pragmatic composite score (formal measures); SC = Social Cognition (formal measure); EF = Executive functions composite score (formal measures); St. lang CCC-2 = A + B + C + D raw scores on CCC-2; Prag CCC-2 = D + E + F + G + H raw scores on CCC-2; Aut CCC-2 = I + J raw scores on CCC-2.

**Table 5:** Summary of regression coefficients for Structural Language CCC-2 scores (A + B + C + D) within the DLD group (bootstrap results based on 2000 bootstrap samples).

Structural Language CCC-2 (A + B + C + D)						
Predictor	R^2^ Adjusted	B	B 95% CI (LL, UL)	SE B	t	*p*
Step 1	0.369					
Constant		51.672	[36.841, 67.801]		6.592	0.001
Age		−0.427	[−0.635, −0.248]	−0.607	−3.972	0.003
Step 2	0.226					
Constant		56.748	[42.854, 70.537]		8.680	0.000
Age		−0.141	[−0.305, 0.149]	−0.200	−1.216	0.167
St. Language		−0.472	[−0.848, −0.208]	−0.626	−3.807	0.013
Step 3	0.026					
Constant		59.296	[44.281, 72.738]		8.804	0.000
Age		−0.137	[−0.292, 0.202]	−0.196	−1.206	0.192
St. Lang		−0.395	[−0.832, −0.162]	−0.524	−2.917	0.031
Prag		−0.907	[−2.346, 0.570]	−0.192	−1.312	0.214
Step 4	0.003					
Constant		59.018	[43.206, 73.028]		8.581	0.000
Age		−0.136	[−0.291, 0.202]	−0.194	−1.176	0.203
St. Language		−0.381	[−0.833, −0.133]	−0.505	−2.685	0.042
Prag		−0.852	[−2.580, 0.661]	−0.181	−1.193	0.300
SC		−0.207	[−1.200, 0.825]	−0.061	−0.430	0.653
Step 5	0.018					
Constant		57.804	[42.260, 72.465]		8.311	0.000
Age		−0.092	[−0.254, 0.323]	−0.131	−0.752	0.453
St. Language		−0.356	[−0.839, −0.109]	−0.472	−2.480	0.060
Prag		−0.568	[−2.315, 1.163]	−0.121	−0.747	0.518
SC		−0.219	[−1.043, 0.838]	−0.064	−0.455	0.648
EF		−0.159	[−0.599, 0.104]	−0.183	−1.061	0.356

Note 1: all are raw scores; Note 2: Age = chronological age (months); St. lang = Language composite score (formal measures); Prag = Pragmatic composite score (formal measures); SC = Social Cognition (formal measure); EF = Executive functions composite score (formal measures).

**Table 6:** Summary of regression coefficients for Pragmatic CCC-2 scores (D + E + F + G + H) within the DLD group bootstrap results based on 2000 bootstrap samples).

Pragmatic CCC-2 (D + E + F + G + H)						
Predictor	R^2^ Adjusted	B	B 95% CI (LL, UL)	SE B	t	*p*
Step 1	0.071					
Constant		34.013	[16.291, 52.549]		3.725	0.003
Age		−0.180	[−0.432, 0.055]	−0.266	−1.435	0.155
Step 2	0.155					
Constant		38.048	[21.054, 55.890]		4.385	0.001
Age		0.048	[−0.282, 0.361]	0.071	0.313	0.755
St. Language		−0.375	[−0.746, −0.003]	−0.518	−2.280	0.037
Step 3	0.060					
Constant		41.751	[21.742, 58.248]		4.703	0.001
Age		0.052	[−0.242, 0.379]	0.078	0.349	0.707
St. Lang		−0.264	[−0.711, 0.053]	−0.364	−1.477	0.138
Prag		−1.318	[−3.045, 0.789]	−0.291	−1.447	0.158
Step 4	0.054					
Constant		40.602	[21.339, 58.148]		4.639	0.001
Age		0.057	[−0.255, 0.357]	0.085	0.387	0.676
St. Language		−0.204	[−0.664, 0.208]	−0.282	−1.130	0.295
Prag		−1.092	[−2.960, 1.203]	−0.241	−1.202	0.295
SC		−0.855	[−2.391, 0.538]	−0.262	−1.395	0.272
Step 5	0.030					
Constant		39.076	[18.758, 56.919]		4.412	0.003
Age		0.112	[−0.191, 0.444]	0.166	0.718	0.433
St. Language		−0.172	[−0.648, 0.191]	−0.238	−0.944	0.377
Prag		−0.735	[−2.722, 1.758]	−0.162	−0.759	0.515
SC		−0.870	[−2.401, 0.461]	−0.266	−1.421	0.252
EF		−0.200	[−0.864, 0.167]	−0.240	−1.047	0.346

Note 1: all are raw scores; Note 2: Age = chronological age (months); St. lang = Language composite score (formal measures); Prag = Pragmatic composite score (formal measures); SC = Social Cognition (formal measure); EF = Executive functions composite score (formal measures).

## Data Availability

The data that support the findings of this study are available from the corresponding author upon reasonable request.

## References

[1] Bishop D.V.M., Snowling M.J., Thompson P.A., Greenhalgh T. (2017). Phase 2 of CATALISE: A multinational and multidisciplinary Delphi consensus study of problems with language development: Terminology. J. Child Psychol. Psychiatry.

[2] Davies C., Andrés-Roqueta C., Norbury C.F. (2016). Referring expressions and structural language abilities in children with specific language impairment: A pragmatic tolerance account. J. Exp. Child Psychol..

[3] Norbury C.F., Nash M., Baird G., Bishop D.V. (2004). Using a parental checklist to identify diagnostic groups in children with communication impairment: A validation of the Children’s Communication Checklist–2. Int. J. Lang. Commun. Disord..

[4] Dockrell J.E., Lindsay G., Palikara O. (2011). Explaining the academic achievement at school leaving for pupils with a history of language impairment: Previous academic achievement and literacy skills. Child Lang. Teach. Ther..

[5] Durkin K., Toseeb U., Botting N., Pickles A., Conti-Ramsden G. (2017). Social confidence in early adulthood among young people with and without a history of language impairment. J. Speech Lang. Hear. Res..

[6] Snowling M.J., Duff F.J., Nash H.M., Hulme C. (2016). Language profiles and literacy outcomes of children with resolving, emerging, or persisting language impairments. J. Child Psychol. Psychiatry.

[7] Bishop D.V.M., Norbury C.F. (2002). Exploring the borderlands of autistic disorder and specific language impairment: A study using standardised diagnostic instruments. J. Child Psychol. Psychiatry.

[8] American Psychiatric Association (2013). Diagnostic and Statistical Manual of Mental Disorders.

[9] Conti-Ramsden G., Botting N. (2008). Emotional health in adolescents with and without a history of specific language impairment (SLI). J. Child Psychol. Psychiatry.

[10] Kilpatrick T., Leitão S., Boyes M. (2019). Mental health in adolescents with a history of developmental language disorder: The moderating effect of bullying victimisation. Autism Dev. Lang. Impair..

[11] Lindsay G., Dockrell J.E. (2012). Longitudinal patterns of behavioral, emotional, and social difficulties and self-concepts in adolescents with a history of specific language impairment. Lang. Speech Hear. Serv. Sch..

[12] Snowling M.J., Bishop D.V., Stothard S.E., Chipchase B., Kaplan C. (2006). Psychosocial outcomes at 15 years of children with a preschool history of speech-language impairment. J. Child Psychol. Psychiatry.

[13] van den Bedem N.P., Dockrell J.E., van Alphen P.M., Kalicharan S.V., Rieffe C. (2018). Victimization, Bullying, and Emotional Competence: Longitudinal Associations in (Pre)Adolescents With and Without Developmental Language Disorder. J. Speech Lang. Hear. Res..

[14] Amador J.A., Forns M., Guàrdia J., Peró M. (2006). Estructura factorial y datos descriptivos del perfil de atención y del cuestionario TDAH para niños en edad escolar [Factor structure and descriptive data from the Attention Profile and the ADHD Questionnaire for school-age children]. Psicothema.

[15] Zhang Y., Xu X., Jiang Y., Sun W., Wang Y., Song Y., Dong S., Zhu Q., Jiang F., Sheng L. (2020). Early language and communication development in Chinese children: Adaptation and validation of a parent report instrument. Int. J. Speech Lang. Pathol..

[16] Thudge J., Hogan D., Greene S., Hogan D. (2004). An ecological approach to observations of children’s everyday lives. Researching Children’s Experience: Approaches and Methods.

[17] Rosetti L. (2006). The Rossetti Infant-Toddler Language Scale.

[18] Rutter M., Bailey A., Lord C. (2003). SCQ: The Social Communication Questionnaire.

[19] Bishop D.V.M. (2003). The Children’s Communication Checklist–2.

[20] Bishop D.V., Norbury C.F. (2005). Executive functions in children with communication impairments, in relation to autistic symptomatology. 1: Generativity. Autism.

[21] Bishop D.V.M. (1998). Development of the Children’s Communication Checklist (CCC): A method for assessing qualitative aspects of communicative impairment in children. J. Child Psychol. Psychiatry.

[22] Bishop D.V.M. (2006). What Causes Specific Language Impairment in Children?. Curr. Dir. Psychol. Sci..

[23] Geurts H.M. (2007). CCC-2-NL: Children’s Communication Checklist-2.

[24] Helland W.A., Biringer E., Helland T., Heimann M. (2009). The usability of a Norwegian adaptation of the Children’s Communication Checklist Second Edition (CCC-2) in differentiating between language impaired and non-language impaired 6- to 12-year-olds. Scand. J. Psychol..

[25] Glumbić N., Brojčin B., Đorđević M. (2010). Pouzdanost Komunikacione čekliste za decu [Reliability of the Communication Checkpoint for Children]. Beogradska Defektološka Škola.

[26] Glumbić N., Brojčin B. (2012). Factor structure of the Serbian version of the Children’s Communication Checklist-2. Res. Dev. Disabil..

[27] Vézina M., Samson-Morasse C., Gauthier-Desgagné J., Fossard M., Sylvestre A. (2011). Développement de la version québécoise francophone du Children’s Communication Checklist—2 (CCC-2): Traduction, adaptation et équivalence conceptuelle. Revue Canadienne d’orthophonie et d’audiologie.

[28] Costa V.B., Harsányi E., Martins-Reis V.O., Kummer A. (2013). Translation and cross-cultural adaptation into Brazilian Portuguese of the Children’s Communication Checklist-2. Codas.

[29] Crespo-Eguílaz N., Magallón S., Sánchez-Carpintero R., Narbona J. (2016). La adaptación al castellano de la Children’s Communication Checklist permite detectar las dificultades en el uso del lenguaje pragmático y diferenciar subtipos clínicos. [The Spanish adapted version of the Children’s Communication Checklist identifies disorders of pragmatic use of language and differentiates between clinical subtypes]. Rev. Neurol..

[30] Mendoza E., Garzón M. (2012). Puede el CCC-2 diferenciar perfiles pragmáticos? [Can CCC-2 differentiate pragmatic profiles?]. Rev. Chil. Fonoaudiol..

[31] Andrés-Roqueta C., Adrian J.E., Clemente R.A., Villanueva L. (2016). Social cognition makes an independent contribution to peer relations in children with Specific Language Impairment. Res. Dev. Disabil..

[32] Bignell S., Cain K. (2007). Pragmatic aspects of communication and language comprehension in groups of children differentiated by teacher ratings of inattention and hyperactivity. Br. J. Dev. Psychol..

[33] Bishop D.V.M., McDonald D. (2009). Identifying language impairment in children: Combining language test scores with parental report. Int. J. Lang. Commun. Disord..

[34] Geurts H., Embrechts M. (2010). Pragmatics in pre-schoolers with language impairments. Int. J. Lang. Commun. Disord..

[35] Grzadzinski R., Di Martino A., Brady E., Mairena M.A., O’Neale M., Petkova E., Castellanos F.X. (2011). Examining autistic traits in children with ADHD: Does the autism spectrum extend to ADHD?. J. Autism Dev. Disord..

[36] Volden J., Phillips L. (2010). Measuring pragmatic language in speakers with autism spectrum disorders: Comparing the children’s communication checklist—2 and the test of pragmatic language. Am. J. Speech Lang. Pathol..

[37] Volden J., Coolican J., Garon N., White J., Bryson S. (2009). Brief report: Pragmatic language in autism spectrum disorder: Relationships to measures of ability and disability. J. Autism Dev. Disord..

[38] Whitehouse A., Barry J.G., Bishop D.V. (2008). Further defining the language impairment of autism: Is there a specific language impairment subtype?. J. Commun. Disord..

[39] Bishop D.V.M., Hardiman M.J., Barry J.G. (2011). Is auditory discrimination mature by middle childhood? A study using time-frequency analysis of mismatch responses from 7 years to adulthood. Dev. Sci..

[40] Dawes P., Bishop D. (2009). Auditory processing disorder in relation to developmental disorders of language, communication and attention: A review and critique. Int. J. Lang. Commun. Disord..

[41] Ferguson M.A., Hall R.L., Riley A. (2011). Communication, listening, cognitive and speech perception skills in children with auditory processing disorder (APD) or Specific Language Impairment (SLI). J. Speech Lang. Hear. Res..

[42] Solomon M., Olsen E., Niendam T., Ragland J.D., Yoon J., Minzenberg M., Carter C.S. (2011). From lumping to splitting and back again: Atypical social and language development in individuals with clinical-high-risk for psychosis, first episode schizophrenia, and autism spectrum disorders. Schizophr. Res..

[43] Quach J., Hiscock H., Canterford L., Wake M. (2009). Australian population longitudinal study: Outcomes of child sleep problems over the school-transition period. Pediatrics.

[44] Hoffmann A., Martens M.A., Fox R., Rabidoux P., Andridge R. (2013). Pragmatic language assessment in Williams syndrome: A comparison of the Test of Pragmatic Language-2 and the Children’s Communication Checklist-2. Am. J. Speech Lang. Pathol..

[45] Ramirez-Inscoe J., Moore D.R. (2011). Processes that influence communicative impairments in deaf children using cochlear implants. Ear Hear..

[46] Andrés-Roqueta C., Katsos N. (2017). The contribution of grammar, vocabulary and theory of mind in pragmatic language competence in children with autistic spectrum disorders. Front. Psychol..

[47] Adams C., Norbury C.F., Tomblin J.B., Bishop D.V.M. (2008). Intervention for children with pragmatic language impairments: Frameworks, evidence and diversity. Understanding Developmental Language Disorders: From Theory to Practice.

[48] Andrés-Roqueta C., Clemente R.A., Aguilar-Mediavilla E., Buil-Legaz L., López-Penadés R., Sanchez-Azanza V.A., Adrover-Roig D. (2019). Idiom understanding competence of Spanish children with Specific Language Impairment. Atypical Language Development in Romance Languages.

[49] Norbury C.F. (2004). Factors supporting idiom comprehension in children with communication disorders. J. Speech Lang. Hear. Res..

[50] Norbury C.F. (2005). The relationship between theory of mind and metaphor: Evidence from children with language impairment and autistic spectrum disorders. Br. J. Dev. Psychol..

[51] Andrés-Roqueta C., Katsos N. (2020). A distinction between linguistic and social pragmatics helps the precise characterization of pragmatic challenges in children with Autism Spectrum Disorders and Developmental Language Disorder. J. Speech Lang. Hear. Res..

[52] Katsos N., Andrés-Roqueta C., Clemente R.A., Cummins C. (2011). Are children with Specific Language Impairment challenged by linguistic-pragmatics?. Cognition.

[53] Surian L., Baron-Cohen S., Van der Lely H. (1996). Are children with autism deaf to Gricean Maxims?. Cogn. Neuropsychiatr..

[54] Brock J., Norbury C., Einav S., Nation K. (2008). Do individuals with autism process words in context? Evidence from language-mediated eye-movements. Cognition.

[55] Norbury C.F., Gemmell T., Paul R. (2014). Pragmatics abilities in narrative production: A cross-disorder comparison. J. Child. Lang..

[56] Andrés-Roqueta C., Ballester-Manuel L., Castejón J. (2016). Comprensión del Humor Gráfico en niños y niñas con Trastorno Específico del Lenguaje y con Trastorno del Espectro Autista [Graphic Humour understanding in children with Specific Language Impairment and children with Autistic Spectrum Disorder]. Psicología y educación: Presente y futuro.

[57] Andrés-Roqueta C., Clemente R.A. (2010). Dificultades pragmáticas en el Trastorno Específico del Lenguaje. El papel de las tareas mentalistas [Pragmatic difficulties in children with specific language impairment. The role of mentalistic tasks]. Psicothema.

[58] Happé F. (1994). An advanced test of theory of mind: Understanding of story characters thoughts and feelings by able autistic, mentally handicapped, and normal children and adults. Autism Dev. Disord..

[59] Botting N., Conti-Ramsden G. (2008). The role of language, social cognition, and social skill in the functional social outcomes of young adolescents with and without a history of SLI. Br. J. Dev. Psychol..

[60] Martin I., McDonald S. (2003). Weak coherence, no theory of mind, or executive dysfunction? Solving the puzzle of pragmatic language disorders. Brain Lang..

[61] Green B.C., Johnson K.A., Bretherton L. (2014). Pragmatic language difficulties in children with hyperactivity and attention problems: An integrated review. Int. J. Lang. Commun. Disord..

[62] Mendoza E., Carballo G., Muñoz J., Fresneda M.D. (2005). CEG: Test de Comprensión de Estructuras Gramaticales [Grammatical Structures Comprehension Test].

[63] Saborit C., Julián J.P. (2005). L’avaluacio del Llenguatge Infantil, ELI [Assessment of Children’s Language].

[64] Conti-Ramsden G., Botting N., Faragher B. (2001). Psycholinguistic markers for specific language impairment (SLI). J. Child Psychol. Psychiatry.

[65] Raven J.C. (1984). Manual for the Coloured Progressive Matrices (Revised).

[66] Norbury C., Bishop D. (2005). Children’s Communication Checklist—2: A validation study. Rev. Tranel (Travaux Neuchâtelois de Linguistique).

[67] Bishop D.V.M., Laws G., Adams C., Norbury C.F. (2006). High heritability of speech and language impairments in 6-year-old twins demonstrated using parent and teacher report. Behav. Genet..

[68] Bishop D.V.M., Maybery M., Wong D., Maley A., Hallmayer J. (2006). Characteristics of the broader phenotype in autism: A study of siblings using the Children’s Communication Checklist-2. Am. J. Med. Genet..

[69] Raven J.C., Court J.H., Raven J. (1990). Section 2: Coloured Progressive Matrices (1990 Edition, with US Norms). Manual for the Raven’s Progressive Matrices and Vocabulary Scales.

[70] Bishop D.V.M. (1983). Test for Reception of Grammar. Medical Research Council.

[71] Kaufman A.S., Kaufman N.L. (2000). K-BIT. Test Breve de Inteligencia de Kaufman (K-BIT).

[72] Wechsler D. (1991). Manual for the Wechsler Intelligence Scale for Children.

[73] Dunn L.M., Dunn L.M., Arribas D. (2006). PPVT-III Peabody, Test de Vocabulario en Imágenes.

[74] Kirk S., McCarthy J., Kirk W. (1968). Illinois Test of Psycholinguistic Abilities. Examiner’s Manual.

[75] Muñoz J., Fresneda M.D., Mendoza E., Carballo G. (2008). Propiedades psicométricas de una prueba de comprensión gramatical [Psychometric properties of a grammatical comprehension test]. Rev. Neurol..

[76] White S., Hill E., Happé F., Frith U. (2009). Revisiting the Strange Stories: Revealing mentalising impairments in autism. Child Dev..

[77] Cairns E., Cammock T. (1978). Development of a more reliable version of the Matching Familiar Figures Test. Dev. Psychol..

[78] Fritz C.O., Morris P.E., Richler J.J. (2012). Effect size estimates: Current use, calculations, and interpretation. J. Exp. Psychol. Gen..

